# Establishment of local diagnostic reference levels for CT colonography at a tertiary hospital

**DOI:** 10.4102/sajr.v28i1.2809

**Published:** 2024-01-31

**Authors:** Filip M. Kozłowski, Christoffel J. van Reenen, Christoph J. Trauernicht

**Affiliations:** 1 Division of Medical Physics, Tygerberg Hospital, Cape Town, South Africa; 2 Department of Medical Physics, Faculty of Medicine and Health Sciences, Stellenbosch University, Cape Town, South Africa

**Keywords:** computed tomography, CT, colonography, dose optimisation, diagnostic reference level, computed tomography dose index-volume, dose-length product

## Abstract

**Background:**

Diagnostic reference levels (DRLs) are an important metric in identifying abnormally high radiation doses in diagnostic examinations. National DRLs for CT colonography do not currently exist in South Africa, but there are efforts to collect data for a national DRL project.

**Objectives:**

This study investigated radiation doses for CT colonography in adult patients at a large tertiary hospital in South Africa with the aim of setting local DRLs.

**Method:**

Patient data from two CT scanners (Philips Ingenuity and Siemens Somatom go.Top) in the period March 2020 – March 2023 were obtained from the hospital’s picture archiving and communication system (PACS) (*n* = 115). Analysis involved determining the median computed tomography dose index-volume (CTDI_vol_) and dose-length product (DLP) values. The findings were compared with DRLs established internationally.

**Results:**

Ingenuity median CTDI_vol_ was 20 mGy and DLP was 2169 mGy*cm; Somatom median CTDI_vol_ was 6 mGy and DLP was 557 mGy*cm. Ingenuity exceeded the United Kingdom’s (UK) recommended DRLs by 82% and 214%, respectively. Somatom median CTDI_vol_ and DLP were 45% and 19% lower than UK NDRLs.

**Conclusion:**

Somatom’s tin filter and other dose reduction features provided significant dose reduction. These data were used to set DRLs for CT colonography at the hospital; CTDI_vol_: 6 mGy and DLP: 557 mGy*cm.

**Contribution:**

In addition to informing radiation protection practices at the level of the institution, the established local DRLs contribute towards implementing regional and national DRLs.

## Introduction

The convenience of using ionising radiation to determine medical diagnoses has led to increased radiation exposure among the general public, with CT being the biggest contributor.^1^ Although radiation exposure from diagnostic imaging carries some risk of carcinogenesis,^1^ the use of radiation in a clinical environment is justified in circumstances where it is more beneficial than harmful to the patient’s well-being.^2^ It is nonetheless imperative to ensure that the dose is as low as reasonably achievable (ALARA), without sacrificing clinical value.

Colorectal carcinoma is the third lethal and fourth most commonly diagnosed cancer in the world, with almost 2 million new cases and 1 million deaths predicted for 2018.^3^ The American Cancer Society estimates 150 000 new cases and 50 000 deaths during 2023 in the United States alone.^4^ Colorectal cancer is highly preventable, with lifestyle changes, physical exercise and screening all leading to lower incidence and mortality.^5^ Computed tomographic colonography, or virtual colonoscopy, is an increasingly common screening method to detect polyps in the colon before they develop into tumours and to identify cancers earlier. Optical colonoscopy remains the gold standard but is invasive and requires anaesthesia for sedation, whereas CT colonography is faster, requires less bowel preparation and has a significantly lower chance of colonic perforation during the procedure.

Justification and optimisation are two key principles outlined by the International Commission on Radiological Protection (ICRP) in Publication 103.^6^ The use of radiation in medicine is justified when the benefits clearly outweigh the harm to the patient. Optimisation is the delicate process of maximising the useful clinical information that is gained from a diagnostic procedure while minimising the harm posed to patients from radiation exposure. Numerous factors can be adjusted to change patient dose, including tube current and voltage and pre-patient beam filtration.^2^ Closely related to justification and optimisation is the ALARA principle, which is at the core of all medical diagnostic examinations involving ionising radiation, and must be enforced wherever possible.

Diagnostic reference levels (DRLs) were first introduced in ICRP Publication 73 as a means of optimisation in diagnostic imaging procedures. Diagnostic reference levels are neither an estimate of patient dose, nor do they represent the maximum allowable dose. Instead, DRLs enable radiation workers to identify situations where patient doses may be unusually high,^7^ ensuring that larger doses are not used when smaller ones can achieve the same clinical outcome with lower risk to the patient. Imaging protocols and radiation protection measures may need to be reviewed if it is found that DRLs are consistently being exceeded, and in practice, this task would involve a multidisciplinary team of radiographers, physicists and clinicians.

In 2017, the ICRP released Publication 135, which expanded upon the previously introduced concept of DRLs. The commission recommends using median values (less influenced by outliers than mean values) when setting DRLs at local and national levels for each imaging modality and clinical procedure.^8^ National diagnostic reference levels (NDRLs) are established by taking the third quartile from the distribution of median values for a specific DRL quantity gathered through a comprehensive nationwide survey of healthcare facilities within a country. The ICRP states that NDRLs should be revised every 3–5 years, with the aim of lowering the levels where feasible, and as technological advancements emerge.

At the time of this publication, NDRLs for diagnostic radiology examinations do not exist in South Africa, and only a few institutions have implemented local DRLs.^9^ According to its annual report for 2019/2020, the National Metrology Institute of South Africa, together with the International Atomic Energy Agency, had set out to establish DRLs for all hospitals in the country that provide diagnostic radiology services.^10^ While that project did not materialise, it is continuing in collaboration with local organisations and institutions, and its scope has been expanded to include all clinical indications in the country (S. Jozela, pers. comm., 24 February 2023). Efforts have been made to aggregate the limited data on DRLs for diagnostic imaging procedures in South Africa;^9^ however, the audit revealed that DRLs have been published for only three types of adult CT examinations, with none pertaining to CT colonography.

Countries such as the United States, Canada, Japan and the United Kingdom (UK) have implemented NDRLs. The UK, in particular, has conducted numerous surveys and reviews since the late 1980s, which have helped with the implementation of NDRLs for a wide variety of clinical indications. In the review conducted in 2011, Shrimpton et al.^11^ suggested a per sequence third quartile computed tomography dose index-volume (CTDI_vol_) value of 11 mGy, and a per complete examination third quartile dose-length product (DLP) value of 950 mGy*cm for CT colonography. In a follow-up review based on data collected between 2017 and 2019, the third quartile DLP value was revised to 690 mGy*cm, with CTDI_vol_ unchanged.^12^ In the same review, the authors compared how the DRL quantities are affected by median and mean values and showed that using the median lead to 8% – 9% lower CTDI_vol_ and DLP values.

### Objectives

The aim of this study was to investigate patient doses from CT colonography examinations through the review of patient data collected from two CT scanners at Tygerberg Hospital and subsequently establish local DRLs.

### Background

Recommended DRL quantities for CT are the CTDI_vol_ and the DLP.^8^ During routine quality assurance and dosimetry procedures, the computed tomographic dose index (CTDI) is measured for a single X-ray tube rotation without table translation, whereby a 100-mm-long pencil ionisation chamber is placed in the centre of a homogeneous cylindrical polymethyl methacrylate (PMMA) phantom and aligned with the CT isocentre. Measurements are obtained inside a phantom; thus CTDI and DLP are machine parameters used to compare treatment protocols, rather than accurate predictors of patient dose during a scan.

CT dose index 100 is the cumulative dose at the centre of a phantom during a 100-mm axial scan,

$$CTDI_{100}=\frac{1}{nT}\int_{-50\;mm}^{+50\;mm}D(z)dz$$ [Eqn 1]

where *D(z)* is the dose profile along the z-axis, *n* is the number of slices acquired per rotation and *T* is the nominal beam width of a single slice (detector row). This gives rise to weighted CTDI (CTDI_w_), which is a good estimate of the weighted average of absorbed dose throughout the phantom and is calculated using dose measurements around the periphery of the phantom and from its centre,

$$CTDI_{w}=\frac{1}{3}CTDI_{100,\text{c}}+\frac{2}{3}CTDI_{100,\text{p}}$$ [Eqn 2]

Volume CTDI (CTDI_vol_) represents the local dose across a single transverse slice during a helical scan and is inherently pitch-corrected. CTDI_w_ from an axial scan can be converted to the equivalent CTDI_vol_ by dividing by the pitch (the ratio of table movement during a single revolution and the beam collimation) of the helical scan,

$$CTDI_{vol}=\frac{CTDI_{w}}{\text{Pitch}}$$ [Eqn 3]

Dose-length product (DLP) approximates the stochastic radiation risk to the patient^13^ and has been shown to give a good estimate of the effective dose during a routine CT scan.^14^ DLP is the product of CTDI_vol_ and the scan length, *L*,

$$DLP=CTDI_{vol}\times L$$ [Eqn 4]

ICRP Publication 135 stipulates that CTDI_vol_ should be reported for each sequence, while DLP should be reported for the entire examination.^8^ The units of CTDI_vol_ and DLP are milligray (mGy) and milligray-centimetres (mGy*cm), respectively.

## Research methods and design

### Design

This investigation was designed as a retrospective study.

### Dosimetry and verification

The two CT scanners at Tygerberg Hospital discussed in this study are the Philips Ingenuity and the Siemens Somatom go.Top. The former was installed in March 2018 and the latter in March 2022. Both scanners were brand new at the time of installation and are covered by a fully comprehensive service contract with the vendors. Each scanner has 128 detector rows and is capable of performing iterative reconstruction, but the key differentiating feature between the two machines is the inclusion of an additional tin filter on the Somatom. As a result, the X-ray beam is hardened, which attenuates the low-energy photons that contribute to patient dose without disrupting any diagnostically useful information. Studies have shown that tin filtration offers significant reduction in patient dose with comparable or improved image quality.^15,16,17,18^

Tube output of both scanners was verified by a physicist using a PTW Nomex dosemeter, a PTW pencil ionisation chamber and a Diagnomatic Pro-CT Dose phantom. The phantom consists of three concentric cylinders corresponding to an adult body (32 cm), adult head and/or paediatric body (16 cm) and paediatric head (10 cm), which can be assembled to form one solid cylinder. Only adult patients were considered in this study, so the complete 32-cm-diameter adult phantom was used to collect measurements. CTDI_vol_ was calculated from the Nomex’s DLP reading using Equation 4, where *L* was the collimated X-ray beam width of a single transverse slice. The physicist confirmed that tube output had not changed since installation.

#### Ingenuity

The Ingenuity’s colonography protocol is a standard abdomen protocol configured for routine administration of iodine-based contrast agents. The protocol consists of a supine and prone scan and one topogram (scout view) for each sequence. Philips Healthcare’s fourth-generation iDose iterative reconstruction algorithm is set to level 4 by default, corresponding to 29% noise reduction compared to filtered back projection^19^ and integrates with the DoseRight dose optimisation software. Tube output was verified with the phantom for each sequence of the clinical protocol.

#### Somatom

The Somatom’s standard low-dose CT colonography protocol is optimised for non-contrast acquisitions and consists of four total scans: supine, prone and two topograms. The protocol employs the CARE Dose4D and CARE kV dose optimisation software, with the latter prioritising a higher level of image quality for the supine scan versus the prone scan. Iterative reconstruction is handled by Siemens Healthcare’s SAFIRE algorithm and defaults to level 3 on the noise reduction scale of 1–5, which represents the middle ground between noisy and smoothed images.^20^ Phantom setup was the same as for the Ingenuity, and tube output was confirmed for the supine and prone sequences.

### Data acquisition and analysis

A total of 115 patients who underwent a CT colonography scan at the hospital during the period March 2020 – March 2023 were considered for this study. Of these, 84 patients were scanned on the Ingenuity (March 2020 – May 2022), and 31 patients were scanned on the Somatom (March 2022 – March 2023). CTDI_vol_ and DLP values for each sequence were collected from the hospital’s picture archiving and communication system (PACS). Data analysis was performed using the Pandas Python package, and the results were compared with existing literature.

### Ethical considerations

Ethics exemption was obtained from the Stellenbosch University Health Research Ethics Committee (HREC) with project ID 26379 and HREC reference number X22/09/022. Only relevant patient data from the hospital’s records were collected and analysed. Due to the retrospective nature of this study, no patient outcomes were directly affected.

## Results and discussion

As recommended by Shrimpton et al.,^11^ CTDI_vol_ was evaluated by taking the average of the supine and prone sequences, whereas DLP was the sum of both sequences. Treating the UK’s NDRLs as a baseline reveals that there are large differences in the dose output of the two scanners. Table 1 shows that the Ingenuity exceeds the UK’s CTDI_vol_ by 82% and the 2019 recommendation for DLP by 214%. The Somatom outperformed the suggested UK limits, with CTDI_vol_ and DLP reading 45% and 19% lower, respectively. Median values are reported for the two scanners (local DRLs), while the UK’s NDRLs represent the 75th percentile of median values of the national distribution. This serves to identify institutions where the median doses are among the highest 25% of the national dose distribution and hence, does not represent optimal doses.^21^

**TABLE 1:** Median *CTDI_vol_ and DLP values compared to NDRLs for CT* colonography in the United Kingdom.

Scanner or Reference	CTDI_vol_ (mGy)	DLP (mGy*cm)
Ingenuity	20	2169
Somatom	6	557
UK (2019)^11,12^	11	690

CTDI_vol_, Computed tomography dose index-volume; DLP, dose-length product.

**FIGURE 1:**
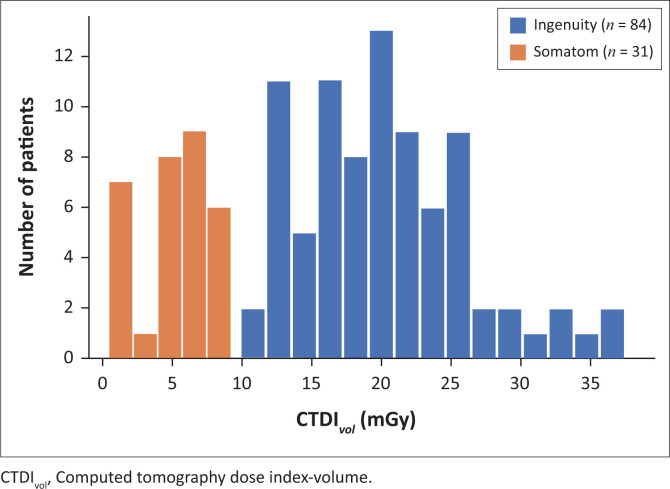
Distribution of median *CTDI_vol_* values.

**FIGURE 2:**
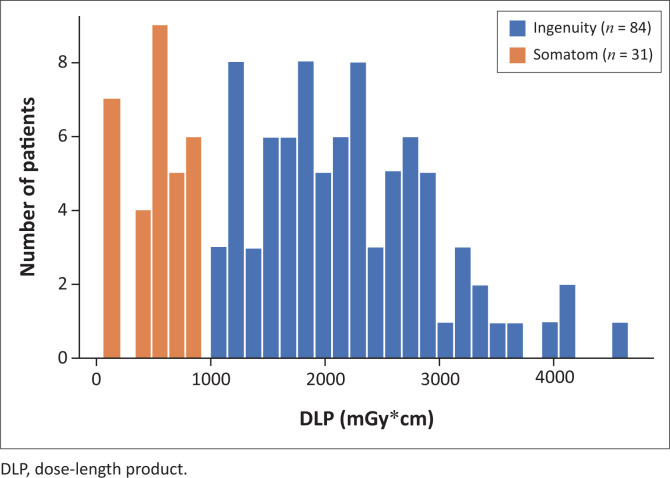
Distribution of median *DLP* values.

The degree to which the Ingenuity exceeds the UK recommendations is exemplified by the distributions of median CTDI_vol_ and DLP values for the two scanners, displayed in Figure 1 and Figure 2, respectively. A noticeable lack of overlap in either distribution emphasises the fact that colonography patients scanned with the Ingenuity receive considerably higher doses than patients scanned with the Somatom. The higher CTDI_vol_ and DLP values cannot be attributed to the degradation of the Ingenuity’s X-ray tube through operational use and ageing because both machines are regularly serviced by accredited technicians. The vendors have implemented their own dose optimisation techniques, and it is apparent that the combination of the Somatom’s tin filter with the SAFIRE algorithm yields significant dose savings.

It is recommended that patients not be scanned on the Ingenuity until such time that stringent optimisation of the scanner’s colonography protocol is performed, in keeping with the ALARA principle. Doing so means that all of the hospital’s colonography patients will be scanned using the Somatom; however, the low number of CT colonography procedures over the most recent 3-year period does not suggest that the scanner’s increased workload will interfere with patient care for other indications. Therefore, local DRLs for CT colonography at Tygerberg Hospital should be set to the values obtained on the Somatom, namely CTDI_vol_: 6 mGy and DLP: 557 mGy*cm.

Given the wealth of data from several comprehensive surveys, the NDRLs for CT colonography adopted in the UK are a good starting point for setting local DRLs in South Africa, where such information is limited. Patient demographics in the UK greatly differ from those in South Africa, especially in public hospitals, which predominantly cater to individuals from rural areas with restricted access to quality healthcare. Nonetheless, the findings demonstrate that established NDRLs can be successfully applied in other countries, provided that dose optimisation is strictly enforced. It is hoped that the NDRL project will produce data for CT colonography and other clinical indications that reflect the South African population.

## Limitations

ICRP Publication 135 recommends collecting data from at least 30 patients when investigating DRLs, and while this criterion was met, the current sample size is nonetheless small. Revisiting this study in the future will likely yield better results because of the availability of more patient data from the Somatom.

The ICRP advocates for the standardisation of patient size by using data from patients that fall within a certain weight range.^8^ It was not possible to do so for this study because patient weight information was not stored in the hospital’s PACS. Moreover, excluding patients outside the weight criteria would have hindered the investigation because of the limited number of patients admitted for CT colonography. The latter issue is especially relevant to the small sample size of Somatom patients.

While NDRL data from multiple countries have been published, information pertaining to CT colonography is scarce. The aforementioned UK reviews stand as the sole sources identified which contain data pertinent to CT colonography; thus, comparing this study’s findings to other national datasets proved to be a challenge.

## Conclusion

Diagnostic reference levels are a useful tool to help reduce radiation doses in patients undergoing diagnostic examinations. Clinical protocols should be adapted to each indication while adhering to the philosophy of keeping patient dose as low as possible without affecting diagnostic quality. Local DRLs for CT colonography were proposed for Tygerberg Hospital, CTDI_vol_: 6 mGy and DLP: 557 mGy*cm, after comparing patient data from two CT scanners with existing literature. Significant dose savings were achieved by using a protocol optimised for CT colonography and a CT scanner equipped with a tin filter. Therefore, recommendations were made to reduce the radiation dose to patients in the future. CT colonography DRL data are lacking both in South Africa and internationally, and the success of the International Atomic Energy Agency project will make a major contribution towards establishing DRLs that are representative of the local population in South Africa.
